# Astrocyte DNA damage and response upon acute exposure to ethanol and corticosterone

**DOI:** 10.3389/ftox.2023.1277047

**Published:** 2024-01-08

**Authors:** Ana Laura Reyes-Ábalos, Magdalena Álvarez-Zabaleta, Silvia Olivera-Bravo, María Vittoria Di Tomaso

**Affiliations:** ^1^ Departamento de Genética, Instituto de Investigaciones Biológicas Clemente Estable (IIBCE), Montevideo, Uruguay; ^2^ Departamento de Neurobiología y Neuropatología, IIBCE, Montevideo, Uruguay

**Keywords:** astrocytes, DNA damage, DDR, ethanol, corticosterone, intercellular communication

## Abstract

**Introduction:** Astrocytes are the glial cells responsible for brain homeostasis, but if injured, they could damage neural cells even deadly. Genetic damage, DNA damage response (DDR), and its downstream cascades are dramatic events poorly studied in astrocytes.

**Hypothesis and methods:** We propose that 1 h of 400 mmol/L ethanol and/or 1 μmol/L corticosterone exposure of cultured hippocampal astrocytes damages DNA, activating the DDR and eliciting functional changes. Immunolabeling against γH2AX (chromatin DNA damage sites), cyclin D1 (cell cycle control), nuclear (base excision repair, BER), and cytoplasmic (anti-inflammatory functions) APE1, ribosomal nucleolus proteins together with GFAP and S100β plus scanning electron microscopy studies of the astrocyte surface were carried out.

**Results:** Data obtained indicate significant DNA damage, immediate cell cycle arrest, and BER activation. Changes in the cytoplasmic signals of cyclin D1 and APE1, nucleolus number, and membrane-attached vesicles strongly suggest a reactivity like astrocyte response without significant morphological changes.

**Discussion:** Obtained results uncover astrocyte genome immediate vulnerability and DDR activation, plus a functional response that might in part, be signaled through extracellular vesicles, evidencing the complex influence that astrocytes may have on the CNS even upon short-term aggressions.

## 1 Introduction

Astrocytes are one of the most abundant glial cells and are responsible for central nervous system (CNS) homeostasis at all levels (Maragakis and Rothstein, 2006; Sofroniew and Vinters, 2010; Verkhratsky et al., 2021). This includes defense, trophic, and energetic support to neurons and oligodendrocytes (Allaman et al., 2011; Wilhelm et al., 2012; Verkhratsky et al., 2021) through one of the most extensive cellular functional repertoires identified (Maragakis and Rothstein, 2006; Sofroniew and Vinters, 2010; Verkhratsky and Nedergaard, 2018; Verkhratsky et al., 2021). However, massive acute or chronic exposure to injuring conditions elicits astrocyte reactivity that includes loss of neuroprotective functions, gain of neurotoxic features, and disturbed cell proliferation that may imply significant alterations in the cell cycle (Sofroniew, 2020; Escartin et al., 2021), leading to significant neural cell damage, which is considered a key factor in the development of several neurological conditions. Many pathological mechanisms implicated in astrocyte contribution to neuronal death have been proposed (Sofroniew and Vinters, 2010; Sofroniew, 2020; Escartin et al., 2021). However, no studies have directly looked at whether astrocytes contribute to genome damage, despite some existing evidence suggesting that astrocyte toxicity might be related to DNA damage (Kok et al., 2021).

**FIGURE 1:**
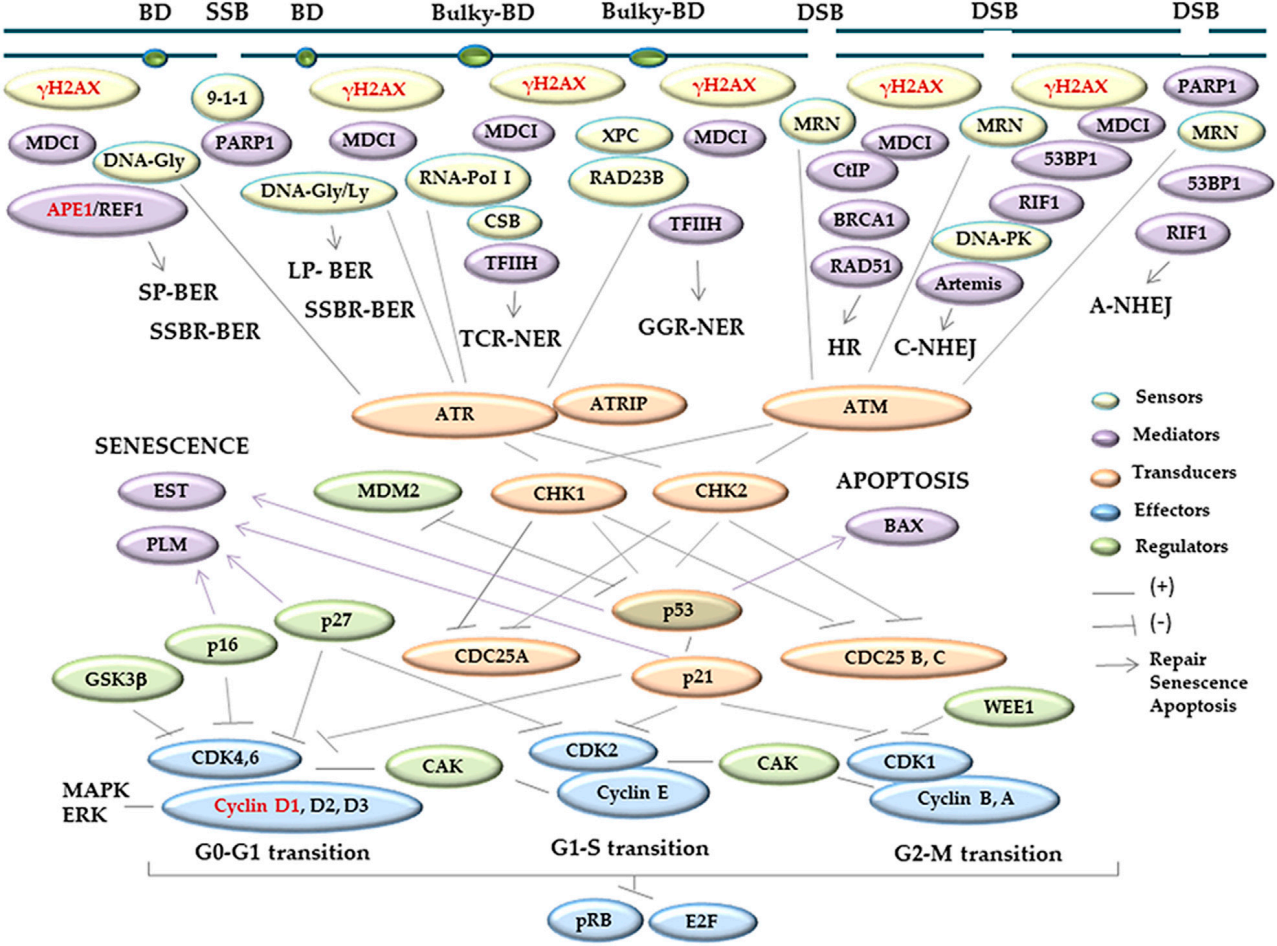
Schematic overview of the major proteins involved in DDR. DDR coordinates different cell pathways, such as cell cycle control, DNA repair, senescence, or apoptosis, setting the fate of the damaged cells. Upon the induction of genetic damage, different proteins are recruited to the DNA damaged sites. There are sensors (yellow) that detect and signalize DNA lesions; mediators (purple) which contribute to the signal and/or link the cell cycle with repair, apoptotic, or senescent pathways, or collaborate with other proteins promoting specific activities; transducers (orange) that help the processing and delivery of the information to the downstream effectors (light blue), which elicit the cellular responses; and regulators (green), which modulate the function of different proteins. The proteins analyzed in the present work are indicated with red letters. The information was obtained from the work of the following authors: Nyberg et al. (2002); Laiho and Latonen (2003); Niida and Nakanishi (2006); Bartek et al. (2007); Huen and Chen (2008); Jackson and Bartek (2009); Koguchi et al. (2002); Musgrove, (2006); and Tchakarska and Sola (2020). Abbreviations ordered as they appear in the scheme: BD, non-bulky base damage, Bulky-BD, Helix-distorting base damage; SSB, single-strand break; DSB, double-strand break; γH2AX, serine-139 phosphorylation of the histone variant H2AX; MDC1, mediator of DNA damage checkpoint protein 1; 9-1-1, recognition complex integrated by RAD9/HUS1/RAD1: RAD9 and HUS1 checkpoint clamp components and RAD1 checkpoint DNA exonuclease, respectively; MRN, recognition complex composed by MRE11/RAD50/NBS1: meiotic recombination11/S.cerevisiae Rad50 homolog double-strand break repair protein/Nijmegen breakage syndrome 1 or nibrin, respectively; DNA-PK, DNA-dependent serine/threonine protein kinase, composed by the catalytic subunit DNA–PKcs and the Ku70 and Ku80 heterodimer; PARP1, [ polyADP-ribosyl] transferase; DNA-Gly, DNA glycosylase with N-glycosylase function; APE1, apurinic/apyrimidinic endonuclease1; REF1, redox-factor 1; DNA-Gly/Ly, DNA glycosylase with N-glycosylase/DNA lyase functions; RNA-PolI, ARN polymerase I; CSB, Cockayne syndrome group B; TFIIH, transcription factor II H; XPC, xeroderma pigmentosum, complementation group C; RAD23B, RAD23 homolog B; CtIP, C-terminal binding protein, CTBP-interacting protein; BRCA1, breast cancer 1; Rad51, *S. cerevisiae* RAD51 homologous recombination protein; 53BP1, p53-binding protein 1; RIF1, replication timing regulatory factor 1; Artemis, NHEJ endonuclease that cleaves 5′ and 3′ overhangs, and hairpins; SP-BER, short-patch route of base excision repair; LP-BER, long patch-base route of base excision repair; SSBR-BER, single-strand break repair by base excision repair; TCR-NER, transcription coupled repair of nucleotide-excision repair; GGR-NER, global genome repair of nucleotide-excision repair; HR, homologous recombination; C-NHEJ, common non-homologous end joining; A-NHEJ, alternative non-homologous end joining rout; ATM, Ataxia telangiectasia mutated; ATR, ATM and Rad3-related; ATRIP, ATR-interacting protein; CHK1,2, checkpoint protein kinases 1 and 2; p53, cellular tumor antigen p53; p21, also known as CDKN1A, cyclin-dependent kinase inhibitor 1A, belonging to the Cip1 family of the CDK-interacting and kinase inhibitory proteins, Cip/Kip; cyclin D1-3, E, B, A/CDK, cyclins/cyclin-dependent kinase complexes; pRB, retinoblastoma-associated protein; E2F, transcription factor E2F; CDK25A,B,C, cell division cycle-25 A, B, C phosphatases; MDM2, murine double minute 2, a E3 ubiquitin ligase protein; GSK-3b, glycogen synthase kinase-3, a proline directed serine-threonine kinase; p16, also identified as CDKN2A, cyclin-dependent kinase inhibitor 2A belonging to the Ink4 family of CDK inhibitors; p27, also known as CDKN1B, cyclin-dependent kinase inhibitor 1B, belonging to the Kip1 family of Kip/Cip CDK inhibitors; WEE1, the Ser/Thr family of protein kinase 1; CAK, CDK-activating kinases; and MAPK/ERK, mitogen-activated protein kinases or extracellular signal-regulated kinases. BAX, pro-apoptotic Bcl-2 antagonist X protein; EST, ever shorter telomeres, a catalytic component of telomerase; and PLM, promyelocytic leukemia protein.

DNA integrity, which is critical for maintaining cells and tissues under physiological parameters, is ordinarily challenged by exogenous and endogenous damaging molecules and also during DNA transcription, replication, and repair (Breen and Murphy, 1995; Friedberg and Wood, 2007). DNA damage is mainly represented by base modifications, some of which can distort the DNA chains and interfere with its replication or transcription, originating single-strand breaks (SSBs) or double-strand breaks (DSB) (Breen and Murphy, 1995; Kawanishi et al., 2006). Cells attempt to solve DNA damage through a complex process known as DNA damage response (DDR). DDR orchestrates cellular mechanisms that elicit the expression of sensor, signaler, transducer, regulator, and effector proteins, which abrogate cell cycle progression at specific sites termed as checkpoints (Nyberg et al., 2002; Laiho and Latonen, 2003; Niida and Nakanishi, 2006; Bartek et al., 2007), (Figure 1). One of the most important sensors of DNA damage is γH2AX, which is formed by the phosphorylation of the nucleosome histone variant H2AX at the 139 serine of the carboxy-terminal tail (Burma et al., 2001; Ward and Chen, 2001) (Figure 1). H2AX phosphorylation occurs at 2 Mbp around the DNA-damaged site, starts a few minutes after DNA damage, reaches the maximum ∼30 min later, and persists up to 3 h after damage repair (Rogakou et al., 1998; Rogaku et al., 1999; Fernandez Capetillo et al., 2003; Kinner et al., 2008). In addition, γH2AX recruits DDR downstream proteins (Koguchi et al., 2002; Laiho and Latonen, 2003; Musgrove, 2006; Niida and Nakanishi, 2006; Bartek et al., 2007; Huen and Chen, 2008; Jackson and Bartek, 2009; Tchakarska and Sola, 2020). Thus, there is a parallelism between the γH2AX signal and the induced DNA damage (Kinner et al., 2008; Amente et al., 2019), which explain that it is a consensus biomarker of DNA damage.

Cyclin D1 is one of the effectors of DNA damage, and its complexes with CDK 4/6 promote the cell cycle progression. However, if these complexes are inhibited, the cell cycle is arrested at the G0–G1 transition checkpoint (Koguchi et al., 2002; Musgrove, 2006) (Figure 1). The DDR cell cycle arrest provides the necessary connections and timing to allow for DNA damage repair (Sancar et al., 2004; Yan et al., 2014; Mjelle et al., 2015) (Figure 1). The apurinic/apyrimidinic endonuclease 1 (APE1) (Figure 1) is a central enzyme in the base excision repair (BER) pathway that restores non-bulky base damage, such as oxidized bases and SSB, limiting secondary DSB production (Caldecott, 2008; Robertson et al., 2009; Kim and Wilson, 2012; Krokan and Bjørås, 2013; Abbotts and Wilson, 2017). APE1 is the main AP-endonuclease in mammalians, and its disordered N-terminus is essential for its recruitment to nuclear subcompartments, including the nucleolus, and the interaction with other BER factors (Tosolini et al., 2020). The expression of APE1 can be induced by oxidative stress, protecting against the genotoxicity of oxidizing agents. Moreover, it is a multifunctional protein with roles in the redox-base activation of transcription factors, including the antioxidant element response among others (Park et al., 2013; Baek et al., 2016). Thus, to orchestrate the cell cycle with DNA repair (Niida and Nakanishi, 2006; Bartek et al., 2007; Huen and Chen, 2008; Jackson and Bartek, 2009), apoptosis (Roos and Kaina, 2006; Larsen and Sørensen, 2017), or senescence (Campisi and d'Adda di Fagagna, 2007; Kuilman et al., 2010) (Figure 1), DDR requires multiple complex and finely tuned protein interactions (Kok et al., 2021).

Among the exogenous compounds that ordinarily challenge DNA integrity are ethanol (EtOH) (Mutlu Türkoğlu et al., 2000; Kido et al., 2001; Russo et al., 2001; Zakhari, 2006) and stress hormones, such as corticosteroids (Madrigal et al., 2001; Flint et al., 2007; Chainy and Sahoo, 2020). Ethanol is one of the most commonly abused drugs, with ∼1.4% of the world’s population having an alcohol use disorder (WHO, 2022). This highly soluble small molecule can cross all biological membranes and barriers, including the blood–brain barrier, and interact via hydrogen bonding and weak hydrophobic interactions with multiple biomolecules (Abrahao et al., 2017). Once absorbed in the body, EtOH is quickly metabolized in many oxidative pathways that may injure DNA on one hand and inhibit the repair of oxidatively damaged DNA on the other (Mutlu Türkoğlu et al., 2000; Zakhari, 2006; Gonthier et al., 2012). Interestingly, stress seems to contribute to increased EtOH consumption (Alhaddad et al., 2020), and the hypothalamic–pituitary–adrenal axis, whose activity produces the adrenocorticotrophic hormone that induces the release of cortisol in humans or corticosterone in rodents (both abbreviated as CTS) (Kadmiel and Cidlowski, 2013), may be involved in alcohol use disorders (Alhaddad et al., 2020). In turn, acute EtOH may activate the hypothalamic–pituitary–adrenal axis, increasing CTS release (Rivier et al., 1984), suggesting a sort of self-reinforcing loop. In addition, CTS could also elicit DNA damage even upon acute, very short *in vitro* exposures (Flint et al., 2007; Flaherty et al., 2017). Moreover, both chronic EtOH and CTS could induce SSB and DSB through an oxidative stress-dependent way (Madrigal et al., 2001; Chainy and Sahoo, 2020) that may affect neuronal survival up to death (Shadfar et al., 2022). The effects of EtOH and/or CTS on DNA damage and repair of glial cells are less known; despite that, it has been proposed that astrocyte DNA damage may contribute to neurodegenerative diseases (Kok et al., 2021).

In the present work, we analyzed whether a short-term exposure of cultured murine hippocampal astrocytes to toxic concentrations of EtOH, in the presence or absence of CTS, could induce DNA DSBs, DDR, and, eventually, astrocyte reactivity. Markers studied by immunoreactivity and confocal imaging were γH2AX, cyclin D1 and APE1, glial acidic fibrillary protein (GFAP), and the calcium-binding protein S100β (Eriksen et al., 2002; Donato et al., 2009; Steiner et al., 2011; Jiménez-Riani et al., 2017; Escartin et al., 2021). This analysis was conducted along with the assessment of the nucleolus number (Pederson, 2011; Weeks et al., 2019) because apart from being classically linked to increased protein synthesis, more recently, it has also been associated with the cellular response to stressors, cell cycle control, DNA replication and repair, and senescence (Pederson, 2011; Weeks et al., 2019). Morphological characterization and the density of membrane vesicles attached to the astrocyte surface were also studied to evaluate the impact of the potential astrocyte reactivity on their signaling that is altered under damaging conditions (Pegtel et al., 2014), including EtOH (Ibáñez et al., 2019). Our findings suggest a significant induction of DNA damage, rapid cell cycle arrest, repair activation, and an astrocyte reactivity-like response that was not accompanied by the typical morphological changes reported in cultured cells (Maragakis and Rothstein, 2006; Sofroniew and Vinters, 2010; Maragakis and Rothstein, 2020; Escartin et al., 2021; Verkhratsky et al., 2021).

## 2 Materials and methods

### 2.1 Animals

In this work, rats from the Wistar lineage (Charles River Laboratory Rats) grown at the School of Science animal house were employed. The animal conditions and procedures were approved by the institutional ethical committee that follows the 18611 National Law for the Use of Animals for Experimental Purposes (N° 005/08/2016). Pregnant rats lived in isolated cages with food and water *ad libitum* and 12 h light/dark cycle at 21°C until delivery. On the fixed day, newborn pups were sexed, and eight were left per mother. Among the remaining rat pups, three males from two different mothers were selected to create astrocyte cultures upon pooling the dissected hippocampi. This procedure was repeated three times to make dose–response curves, and it was repeated another seven times to make seven independent cultures and experiments with the selected working concentrations. In total, we employed 30 male Wistar rats of 1 postnatal day that came from at least 20 different mothers to avoid the litter effect (Jiménez and Zylka, 2021). Males were selected because of the sex differences in response to corticoid hormones (De Nicola et al., 1998; García-Cáceres et al., 2010) and alcohol-induced neurotoxicity (Wilhelm et al., 2015). Future experiments including both males and females are planned.

### 2.2 Hippocampal primary astrocyte cultures

Seven independent cultures were performed with three rat pups per culture. The protocol was carried out according to the work of Olivera-Bravo et al. (2011) with minor modifications. In brief, rat pups were decapitated under a laminar flow hood, each brain was dissected and placed in sterile 10 mmol/L, pH 7.4 PBS, and the meninges were removed. The clean brain was transferred to another plate with sterile PBS, where the hippocampi were dissected and pooled. Small hippocampus pieces were incubated in 0.5% Trypsin-EDTA for 25 min in a 37°C water bath with gentle agitation. Trypsin was then blocked with DMEM-10% fetal bovine serum (FBS, Gibco, 12657011), and repeated pipetting was carried out until tissue homogenization. The homogenate was passed through a sterile 80 µm sieve and centrifuged at 400 *g*, and the resulting pellet was diluted in 5 mL of DMEM medium (modified Eagle’s medium, Gibco, 12800082, HEPES and NaHCO_3_) supplemented with 10% FBS and 1% penicillin/streptomycin antibiotic mixture (Gibco, 15140122). Cells were seeded in culture bottles with a filter cap (T25, Eppendorf) at a density of 400,000 cells/mL and incubated at 37°C with 95% O_2_/5% CO_2_. The culture medium (CM) was changed three times a week until confluence. At this time, cells were shaken during 48 h to enrich the culture in astrocytes and then left to rest for a week. Then, the CM was completely retired, and 0.5% trypsin was added during 5 min and incubated at 37°C. The trypsin-blocked cells were spun at 400 g and were counted and re-seeded on different substrates depending on the test to be performed. Teflon slides with eight wells of 6 mm diameter (Tef-Tek Micro Slides Premium, PorLab), 4 mm diameter glass coverslips (CitoglasR) for immunostaining and fluorescence microscopy, or autoclaved Aclar film (Aclar, Electron Microscopy Sciences) for scanning electron microscopy were used. 24 h before each experiment, the percentage of FBS was decreased to 2% to favor the quiescence of the culture (Olivera Bravo et al., 2011; 2015).

### 2.3 Culture treatments

**FIGURE 2:**
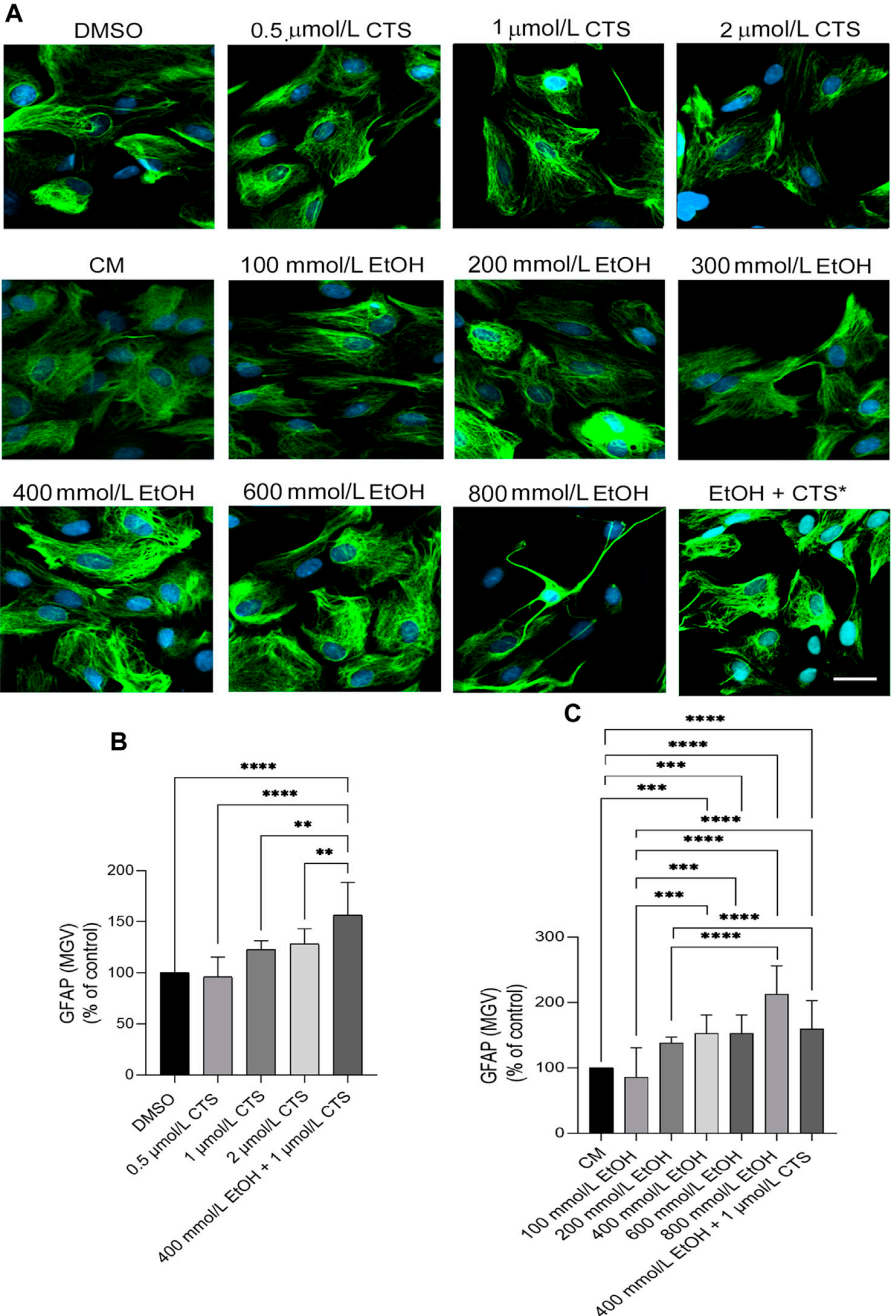
GFAP immunoreactivity upon EtOH or CTS increasing concentrations. **(A)** Confocal images of the GFAP signal (green) after 1 h of incubation with 0–2 μmol/L CTS or 0–800 mmol/L EtOH revealing the absence of significant morphological changes except at 800 mmol/L. Nuclei were labeled with DAPI (blue). Calibration bar: 10 μm. **(B,C)** GFAP MGV determined at different CTS **(B)** or EtOH **(C)** expressed as the percent of respective controls, indicating significant increases along with an increase in EtOH and/or CTS concentrations. In this and all the figures, bars show the median with 95% confidence interval. Only the statistically significant comparisons at *p < 0.05* were represented. Number of asterisks indicates *p-values* lesser than *0.05* (*), *0.01* (**), *0.001* (***), or *0.0001*(****). A total number of 150 cells per condition were analyzed in three separate experiments.

**FIGURE 3:**
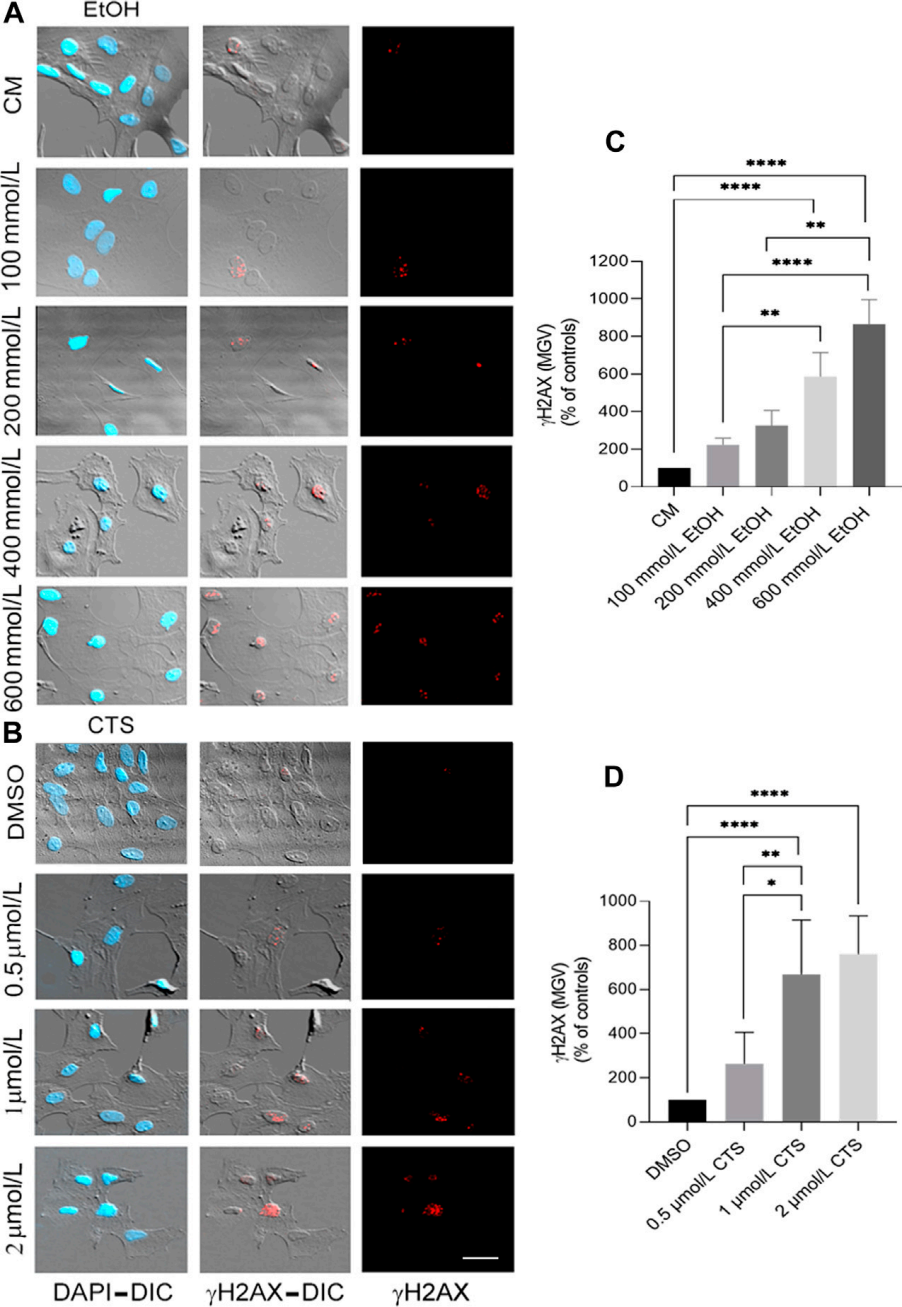
Primary DNA damage induced by EtOH and/or CTS increasing concentrations. **(A, B)** Representative DIC images and γH2AX immunofluorescence (red) elicited by 0–800 mmol/L EtOH **(A)** or 0–2 μmol/L CTS **(B)** after 1 h incubation revealing the presence of chromatin γH2AX foci. Nuclei were labeled with DAPI (blue). Calibration bar: 10 μm. **(C,D)** MGV of γH2AX foci at different CTS **(C)** or EtOH **(D)** concentrations evidencing a concentration-dependent response. As in Figure 2, only statistical differences were represented. A total number of 250 cells per condition were analyzed in three independent experiments.

**FIGURE 4:**
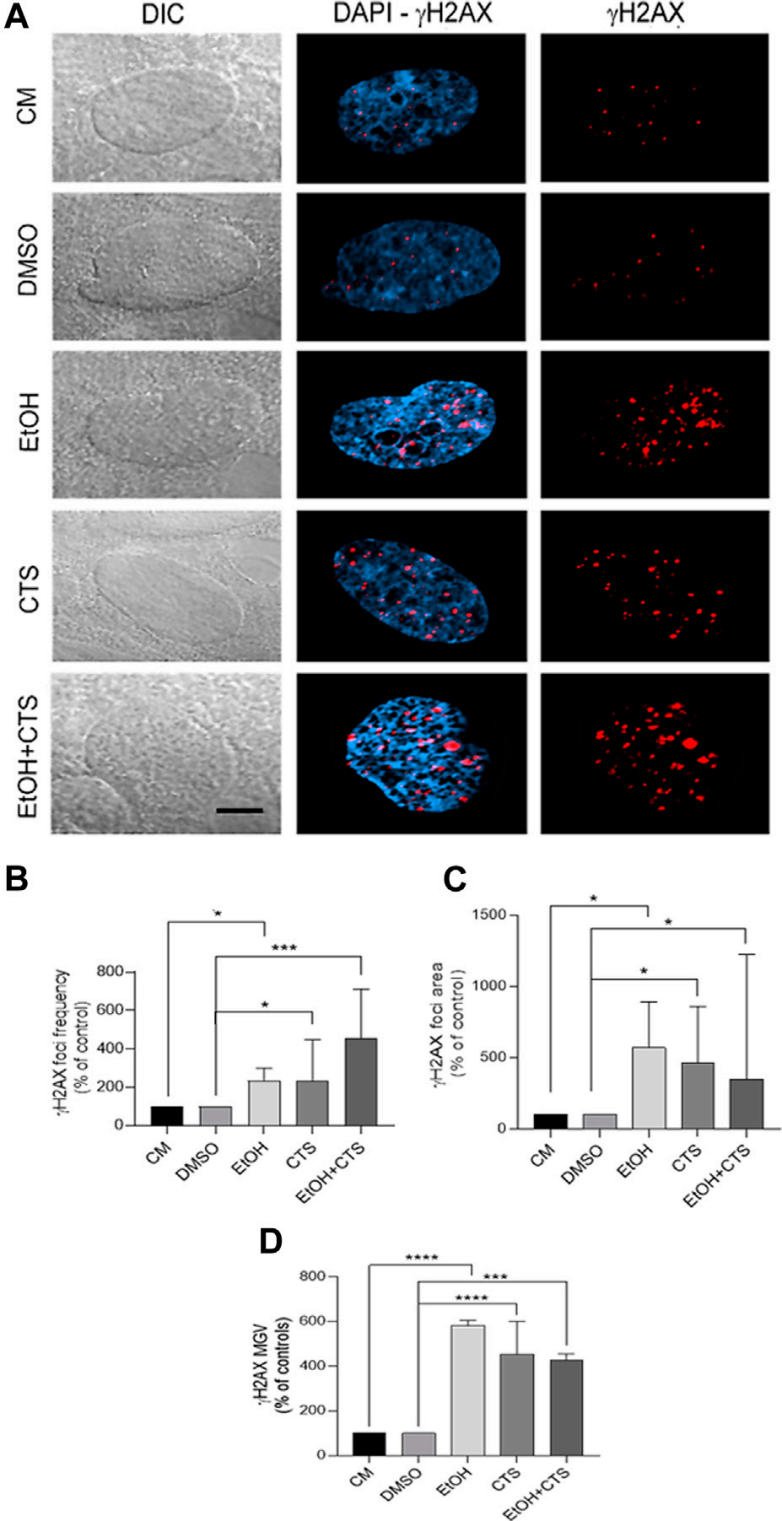
Primary DNA damage induced by 400 mmol/L EtOH and/or 1 μmol/L CTS. **(A)** DIC and confocal images evidencing the sites of nuclear DNA damage recognized as γH2AX foci (red). Nuclei were labeled with DAPI (blue). Calibration bar: 5 μm. **(B,D)** Frequency **(B),** area **(C),** and MGV **(D)** of γH2AX foci expressed as the percentage of controls. In all the parameters analyzed, EtOH, CTS, and EtOH + CTS exposures induced significant increases related to controls but no differences among them. At least 250 cells per condition were analyzed in seven independent experiments.

To select the EtOH and CTS working concentration, dose–response curves were made by adding 0–800 mmol/L EtOH or 0–2 μmol/L CTS to confluent astrocyte cultures during 1 h. Astrocyte morphology, number, and survival was assessed in the DIC and confocal images of GFAP immunostaining (Figure 2) and phalloidin labeling (Jiménez-Riani et al., 2017; Supplementary Figure S1). DNA damage was assessed by γH2AX immunofluorescence (Figures 3, 4).

Based on the dose–curve analysis, the experimental conditions fixed were the following: controls (CM and 0.03% DMSO), EtOH (400 mmol/L, Šarc and Lipnik-Stangelj, 2009), corticosterone (CTS, ab143597, 1 μmol/L, Chatterjee and Sikdar, 2013), and EtOH + CTS (400 mmol/L and 1 μmol/L), respectively. To set the experiments, bleomycin (2.5 μg/L) was initially used as a positive DNA damage control (Liddle et al., 2014). All treatments lasted 1 h. Upon this time, cells were immediately washed in PBS and fixed with 4% paraformaldehyde (PFA, 15 min). After three washes with PBS (10 min each), cells were submitted to indirect immunofluorescence.

### 2.4 Indirect immunofluorescence

Fixed cells were washed in PBS, three times during 3 min each, permeabilized with 0.5% Triton X-100 (20 min), and nonspecific binding was blocked with 2% bovine serum albumin (BSA, 30 min). Next, incubation with primary antibodies (Table 1) diluted in 2% BSA at 37°C for 30 min in a humid chamber was carried out. After that, cells were washed three times (5 min each) with PBS, and the incubation with secondary antibodies conjugated with 488, 546, or 633 nm fluorophores lasted 30 min at 37°C in a humid and dark chamber. In the set of dose–response experiments, 1:250 dilutions of Alexa Fluor™ 633 phalloidin (A22284, Invitrogen) were added together with the secondary antibodies. After three washes (5 min each), cells were incubated with 1.5 mg/L 4.6-diamidino-2-phenylindole (DAPI, ab2629482, Abcam) that was used as the nuclear label. Then, the cells were mounted in ProLong Gold antifade (P36930, Invitrogen) and sealed with colorless nail enamel (Liddle et al., 2014; Reyes-Ábalos et al., 2018). The preparations were protected from light and preserved at 4°C until confocal microscopy imaging.

**TABLE 1:** Antibodies employed in this work.

Primary antibodies (name, manufacturer, code)	Dilution
Rabbit Anti-GFAP antibody, Sigma, G9269	1/400
Mouse Anti-gamma H2AX (phospho S139) antibody [9F3], Abcam, ab26350	1/300
Rabbit Anti-Cyclin D1 antibody, Abcam, ab16663	1/200
Mouse Anti-APE1 antibody, Abcam, ab194	1/500
Mouse Anti-S-100 (β-Subunit) Protein antibody, Sigma-Aldrich, S2532	1/500
Rabbit Anti-Histone H3 (trimethyl K4) antibody - ChIP Grade, Abcam, ab8580	1/400
Mouse Anti-Histone H3 (trimethyl K27) antibody - ChIP Grade, Abcam, ab6002	1/200
Rabbit Anti-RNP antibody, Kun. et al. (2007)	1/500
Secondary antibodies (name, manufacturer, code)	Dilution
Goat anti-mouse IgG AlexaFluor 546, Invitrogen, A-11030	1/300
Goat anti-rabbit IgG AlexaFluor 488, Invitrogen, A-11008	1/500
Goat anti-chicken IgY (H + L) Alexa Fluor™ 488, Invitrogen, A-11039	1/500
Goat anti-chicken IgG H + L AlexaFluor 633, Invitrogen, A-21103	1/300

A summary of the primary and secondary antibodies employed in this work is provided. The presented information indicates the species in which each antibody was developed, the manufacturer, code number, and dilution employed. The immunocytochemistry protocol used is detailed in Materials and Methods.

### 2.5 Confocal microscopy

The cells of the different experimental groups and independent experiments were imaged in a Zeiss Laser Scanning Microscope (LSM-800 that has three GaAsP detectors, a T-PMT detector, and four laser lines: 405, 488, 561, and 640 nm). Acquisition was made by using a Plan Apochromatic oil immersion lens (63×, 1.4 NA) with and without zoom in sequential scans at 2,048 × 2,048 pixels and using the necessary laser lines. Acquisition parameters were preserved among all the groups of each experiment. All conditions from each experiment were imaged on the same day.

### 2.6 Scanning electron microscopy

The procedure applied was adapted from the work of Heckman et al. (2004). Astrocytes cultured on Aclar film were fixed in warm glutaraldehyde 2.5% in 100 mmol/L, pH 7.3 phosphate buffer (PB) for 18 h, then removed from the fixative solution, and washed three times in PB (5 min each). Then, a 30 min post-fixation treatment with osmium tetroxide to stabilize cell membranes was carried out. After five washes of 10 min each, a chemical dehydration based on increasing EtOH concentrations (50%, 70%, 80%, 90%, and 100%) was carried out. Subsequently, the astrocytes were subjected to the elimination of solvents using drying equipment at a CO_2_ critical point to maintain the cellular internal structure intact. Finally, metallization with pure gold through a sputtering technique (gold plasma) and mounting in individual bronze dowels was carried out. Samples were observed in a Jeol JSN5900 LV scanning electron microscope (Scanning Electron Microscopy Unit, School of Sciences, University of the Republic). Images of the astrocyte surface of each experimental condition were obtained using secondary electrons at 20 mA with ×1,000, ×2,000, ×3,000, ×10,000, and ×30,000 magnifications and saved in non-compressed (.TIFF) format (Heckman et al., 2004).

### 2.7 Image analysis

Confocal digitized images were analyzed using the FIJI (NIH) software to determine the number of cells or foci per image using the cell counter plugin. The intensity per pixel number (mean gray value, MGV) and area of each marker analyzed were measured using the ROI and Measure Tools. The density and appearance of extracellular vesicles attached to the surface of astrocytes scanned by SEM were determined using the same software and tools.

### 2.8 Statistical analysis

At least five independent experiments were conducted, each one per triplicate or quintuplicate per condition. Around 150–250 cells per condition were analyzed per marker studied and each set of experiments; all distinguishable vesicles attached to the astrocyte surface were counted, and their main morphological parameters were identified. Data were analyzed with GraphPad Prism 8.3. The Shapiro–Wilk test was applied to check the normal distributions of the variables. Comparisons among groups were carried out with the non-parametric Kruskal–Wallis test, followed by Dunn’s multiple comparison tests. Odds ratio calculation (OR, library “epiR” and “MASS”) between an exposure (EtOH and/or CTS) and the generated genetic damage (foci of γH2AX), its 95% confidence interval (CI), the standard error, and the *p*-value were calculated using Fisher’s exact text.

Colocalization of γH2AX foci and cyclin D1 was achieved employing M1 (co-occurrence between γH2AX and cyclin D1 signals) and M2 (co-occurrence between cyclin D1 and γH2AX signals) (Manders et al., 1993; Aaron et al., 2018). Manders and rho and tau correlation coefficients were available in the FIJI software (Coloc 2). Colocalized pixel maps between γH2AX and cyclin D1 signals were obtained using the Colocalization Threshold plugins of FIJI. In all the cases, the statistical significance level was determined at *p < 0.05*. The number of asterisks indicate *p-values* less than 0.05 (*), 0.01 (**), 0.001 (***), and 0.0001 (****). Only the statistically significant comparisons (*p < 0.05*) are shown in the charts.

## 3 Results

### 3.1 DNA damage and DDR activation upon EtOH and/or CTS incubation

Confluent cultures of hippocampal astrocytes maintained in DMEM–2% FBS for 24 h were incubated with 0–2 μmol/L CTS or 0–800 mmol/L EtOH during 1 h to analyze astrocyte morphology (Figure 2) and determine the minor concentrations in which astrocytes suffer evaluable DNA damage in the chromatin context (Figure 3). Analysis of GFAP immunoreactivity at all the concentrations employed revealed increases in the intensity per area (MGV) but an absence of clear signs of reactivity, except at 800 mmol/L EtOH, in which few astrocytes clearly show cell-body shrinkage and long cell processes (Figure 2). Statistical analysis of GFAP data obtained from the dose–response curve indicates values of Kruskal–Wallis statistics of 95.58 and 35.93 for EtOH and CTS, respectively; *p < 0.0001* in both cases.

Concerning the analysis of the sensor of DNA damage employed, significant and specific γH2AX immunoreactivity were observed at 1 μmol/L CTS and 400 mmol/L EtOH (Figure 3). As accompanying DIC images and Alexa 633-phalloidin labeling (Supplementary Figure S1) indicate that astrocytes do not show significant signs of cell death or detachment from the substrate, both concentrations were selected as the working concentrations. Statistical analysis of γH2AX data obtained from the dose–response curve indicates values of Kruskal–Wallis statistics of 59.66 and 41.64 for EtOH and CTS, respectively, with *p < 0.0001* in both cases.

Once 400 mmol/L EtOH and/or 1 μmol/L CTS were determined as the working concentrations, upon 1 h exposure, a significant immunoreactivity against γH2AX appeared as nuclear aggregates (foci, red) (Figure 4A) that are more abundant and with higher area and intensity compared to the controls (Figures 4B–D) but similar among the co-exposure and each individual noxa. Statistical analysis for EtOH, CTS, and EtOH + CTS groups compared to each respective control indicates *p-values* of *0.0212, 0.0340,* and *0.0005* for focus frequency, *0.0286, 0.0280,* and *0.0270* for area, and *<0.0001, <0.0001,* and *0.0005* for intensity. Kruskal–Wallis statistics for frequency, area, and MGV of γH2AX foci were 32.57, 14.96, and 15.55, respectively, whereas the respective *p-values* were *p < 0.0001, p = 0.0048*, and *p = 0.0037.* In addition, the probability of astrocytic damage as assessed by the odds ratio (OR) (with range and *p-values*), to evaluate the presence and absence of γH2AX foci in all conditions, indicates a greater probability of damage associated with EtOH and/or CTS challenges than in its absence (Table 2).

**TABLE 2:** Odds ratio of treated vs. control astrocytes.

Experimental condition	OR	95% CI	*p*-value
EtOH vs. CM	2.53	1.94–3.29	3.94 e^−12^
CTS vs. DMSO	1.48	1.16–1.89	0.00162
EtOH vs. CTS	1.90	1.43–2.52	9.4 e^−6^
EtOH + CTS vs. EtOH	1.80	1.35–2.43	5.14 e^−5^
EtOH + CTS vs. CTS	2.80	2.12–3.69	1.2 e^−13^
EtOH + CTS vs. DMSO	4.15	3.17–5.43	<2.2 e^−16^

Odds ratio and 95% confidence interval values evidencing that the probability of astrocyte damage elicited by EtOH and/or CTS exposure was greater than in the absence of such treatments. Values were obtained from the analysis of the damage sensing marker γH2AX for all the experimental conditions assessed as the number of nuclei with foci vs. the number of nuclei without foci. Statistical analysis and tests employed are detailed in Materials and Methods.

**FIGURE 5:**
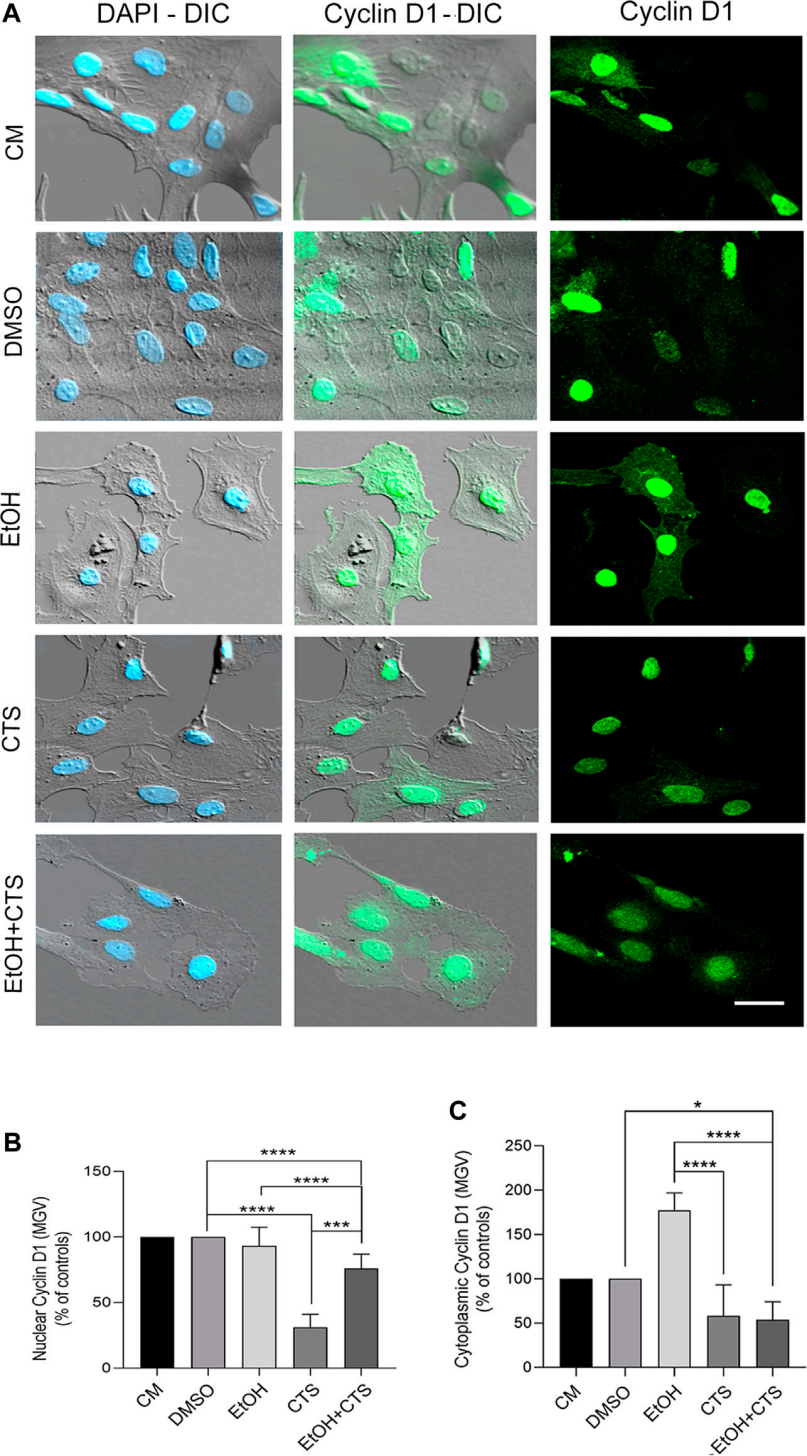
Changes in cyclin D1 immunoreactivity after EtOH and/or CTS exposure. **(A)** DIC and confocal images of nuclear and cytoplasmic cyclin D1 (green) in all experimental conditions. The higher nuclear intensity and appearance as aggregates of different sizes and intensities should be noted. Nuclei were labeled with DAPI (blue). Calibration bar: 10 μm. **(B,C)** MGV of nuclear and cytoplasmic cyclin D1 in all experimental conditions. Regarding nuclear cyclin D1, there were no changes upon EtOH but significant reduction in CTS and intermediate values in the EtOH + CTS condition. Cytoplasmic cyclin D1 MGV did not change in individual exposures, but decreased in EtOH + CTS. At least 250 cells per condition were analyzed in five independent experiments.

Concerning cyclin D1 immunoreactivity**,** after the exposure to EtOH and/or CTS, it presented different intensities in the nuclear and cytoplasmic compartments (green, Figure 5A). The assessment of MGV of the nuclear cyclin D1 expressed as the controls’ percent indicates that while the EtOH group did not differ from the controls (*p = 0.9580*), the CTS MGV was the minor (*p < 0.0001* related to controls) and that EtOH + CTS showed an intermediate value among each separated noxa (*p < 0.0001*; Figure 5B). Remarkably, the MGV of cytoplasmic cyclin D1 relative to controls indicates that the EtOH group showed the maximal values when compared with CTS and EtOH + CTS (*p < 0.0001* in both cases) and that CTS and EtOH + CTS were similar (*p = 0.1015*; Figure 5C). DIC images clearly evidence that signals were restricted to the nucleus and throughout the whole cell body without significant background or unspecific binding. Statistical analysis for EtOH, CTS, and EtOH + CTS compared to each respective control revealed *p-values* of *0.9580, <0.0001,* and *<0.0001* for the nuclear MGV and *0.1631, 0.1015* ***,*** and *0.0348* for the cytoplasmic signal. The Kruskal–Wallis statistics for nuclear and cytoplasmic cyclin D1 were 106.9 and 90.85, whereas *p < 0.0001* was the *p-values* in both cases.

Interestingly, the γH2AX foci (red) and nuclear cyclin D1 (gray) immunoreactivities colocalized, and the overlapped areas increased in EtOH, CTS, and EtOH + CTS compared with the controls, as shown in the colocalization pixel maps for each condition (Supplementary Figure S2, left and right images), but did not differ among them. Values of Manders (Supplementary Figures S2B, C) and rho and tau (Supplementary Figure S2D) correlation coefficients indicate increases in EtOH and CTS and a tendency to increase in EtOH + CTS, compared to controls, but no differences among these three groups.

**FIGURE 6:**
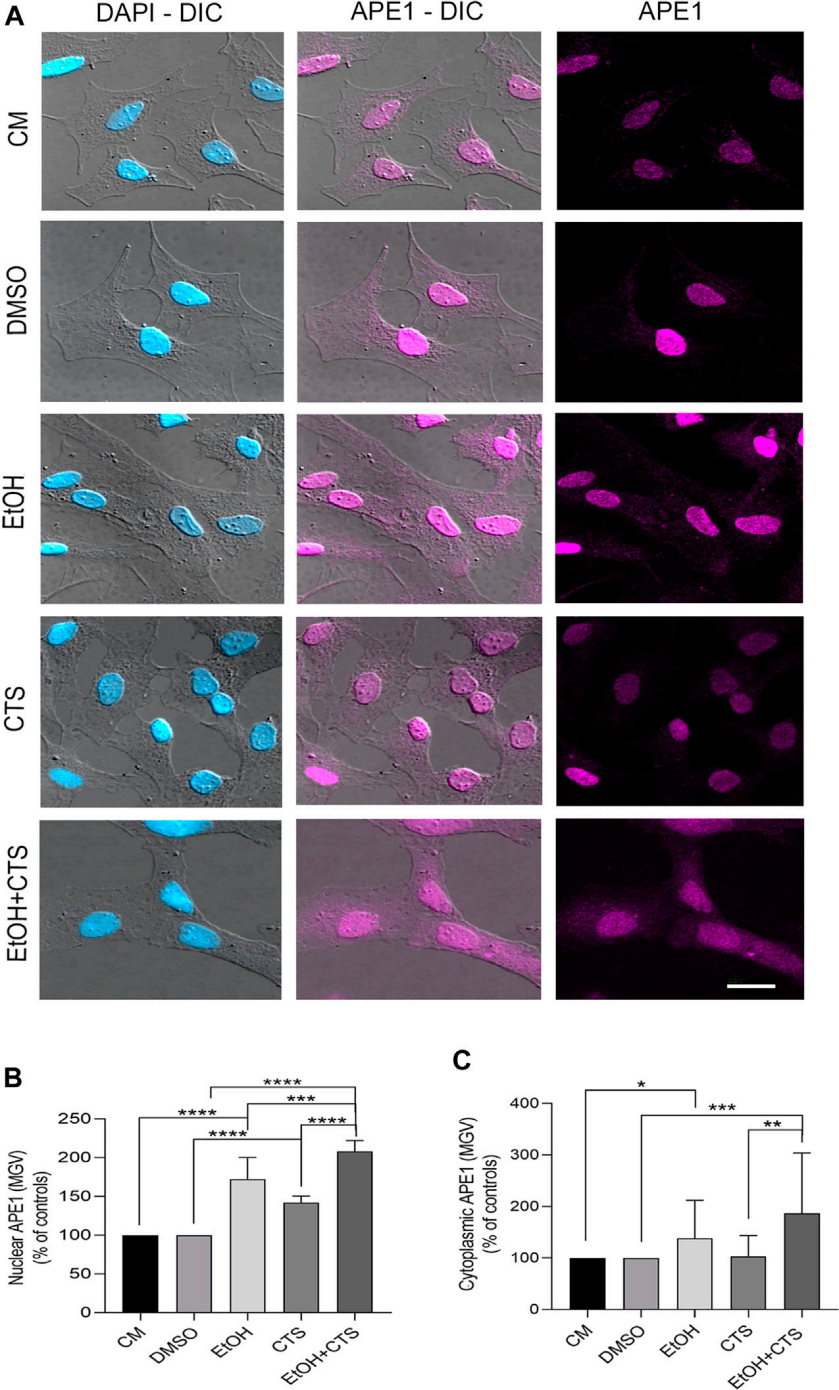
APE1 levels suggest DNA repair and downstream anti-inflammatory response to EtOH and/or CTS treatment. **(A)** DIC and confocal images showing nuclear and cytoplasmic APE1 signals (fuchsia) in all experimental conditions. The clear prevalence of nuclear staining that appears as small aggregates should be noted. Calibration bar: 10 µm. **(B,C)** Nuclear and cytoplasmic APE1 MGV in all experimental groups. Significantly increased values in nuclear APE1 were found in EtOH, CTS, and EtOH + CTS conditions, with the co-exposure being higher than EtOH or CTS alone. Cytoplasmic APE1 MGV was significantly higher than in controls in all conditions with EtOH, and EtOH + CTS show the highest values. At least 250 cells per condition were analyzed in five independent experiments.

Regarding APE1 immunoreactivity, it was positive in both the nucleus and the cytoplasm in all the experimental conditions, but the nuclear signal predominated over the cytoplasmic one (magenta, Figure 6A). The analysis of APE1 nuclear MGV parametrized to the controls evidenced values significantly higher in EtOH, CTS, and EtOH + CTS than in the controls (*p < 0.0001* in all cases), and the co-exposure was higher than each separated noxa (*p = 0.0029* and *p < 0.0001* for EtOH and CTS, respectively) (Figure 6B). Regarding the MGV of cytoplasmic APE1, it was significantly higher in EtOH (*p = 0.0145*) and EtOH + CTS (*p = 0.0004*) than in the controls, and it was also significantly higher in EtOH + CTS than in CTS alone (*p < 0.000*1), (Figure 6C). The Kruskal–Wallis statistics for nuclear and cytoplasmic APE1 were 150.8 and 130.5, with *p < 0.0001* in both cases.

### 3.2 Astrocyte reactivity-like upon EtOH and/or CTS

**FIGURE 7:**
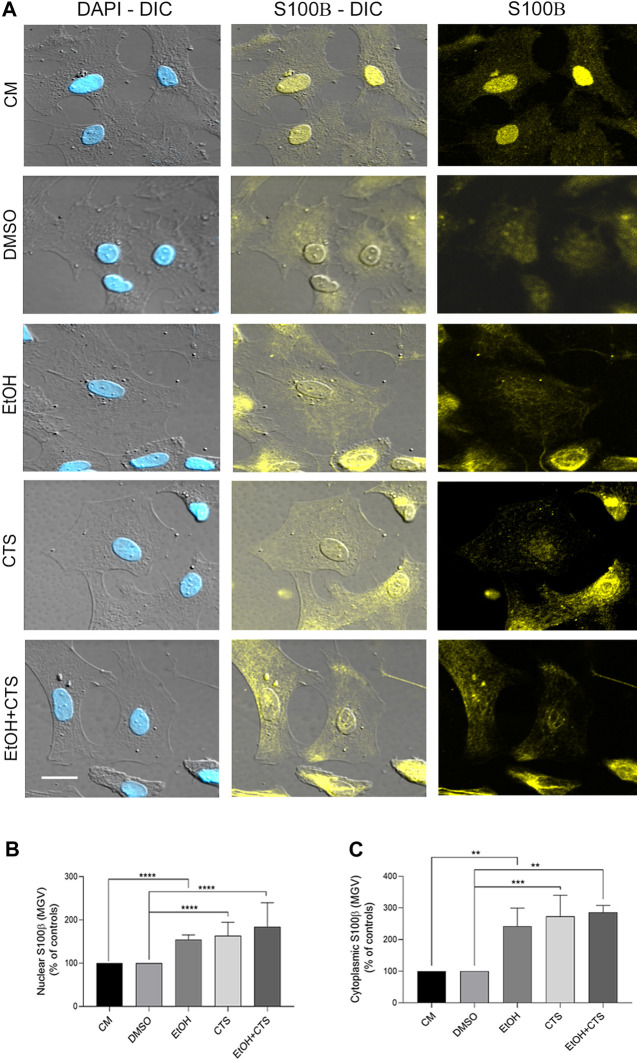
S100β immunoreactivity elicited by EtOH and/or CTS challenge. **(A)** DIC and confocal images of individual cells showing nuclear and cytoplasmic S100β (yellow) signals alone or merged with the nuclear staining DAPI (blue). Calibration bars: 10 µm. **(B,C)** S100β MGV in the nucleus **(B)** and the cytoplasm **(C)** showing higher values upon EtOH and/or CTS exposures. In both the nucleus and cytoplasm, the MGV of EtOH and/or CTS was significantly higher than in controls and without differences with EtOH + CTS. At least 250 cells per condition were analyzed in five independent experiments.

In addition to the analysis of GFAP immunoreactivity performed during dose–response experiments (Figure 2), S100β immunoreactivity was evaluated upon 400 mmol/L EtOH and/or 1 μmol/L CTS challenges. This immunolabeling was made to assess the astrocyte response linked not only to morphological changes but also to downstream damaging cascades. As found with GFAP, no detectable changes in morphology or cell number were found in any condition (Figure 7A). However, images obtained show higher nuclear signals and significant cytoplasmic localization in all experimental conditions. Accordingly, increased nuclear and cytoplasmic MGV were determined in EtOH, CTS, and EtOH + CTS groups related to controls (*p < 0.0001* in all cases) but without differences among each of these groups (Figures 7B,C). The Kruskal–Wallis statistics for nuclear and cytoplasmic S100β were 108.1 and 119.9, respectively, with *p < 0.0001* in both cases.

**FIGURE 8:**
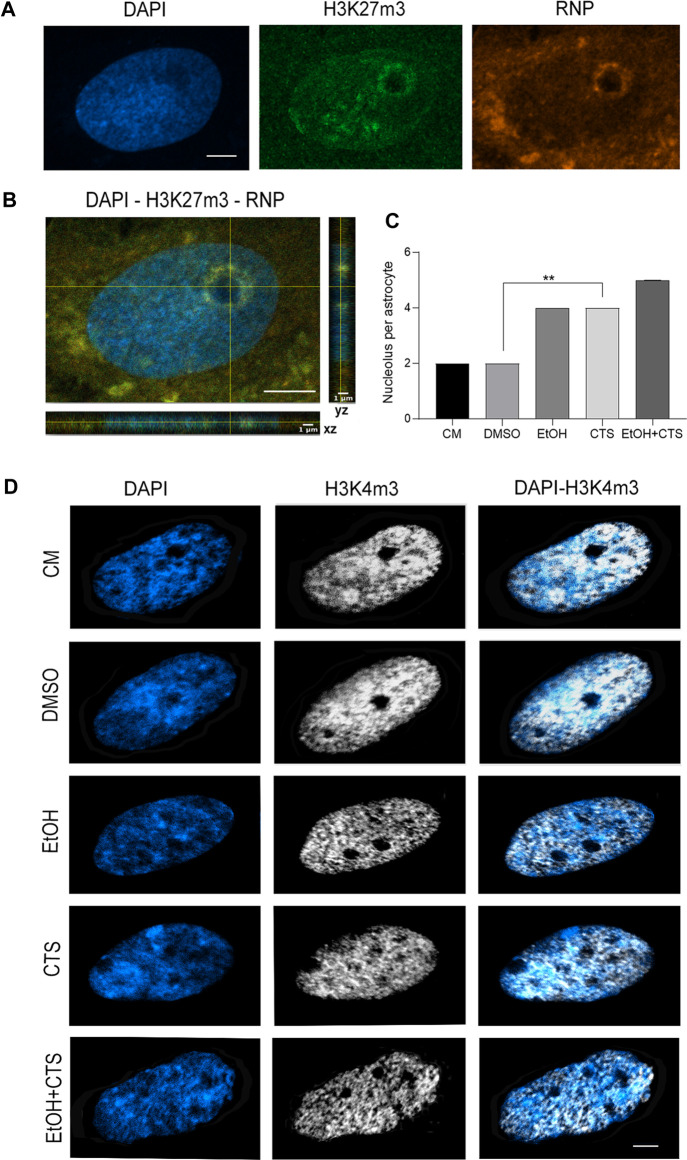
Increased nucleolus number per cell upon CTS incubation. **(A)** Visualization of the nucleolar domain recognized as a non-stained DAPI region surrounded by a region DAPI-intense with positive signals of the heterochromatin marker (H3K27m3, green) and co-labeled with rARN and ribosomal proteins (red). Calibration bar: 5 µm. **(B)** XY and ZX cutting planes of confocal images pointing to an intra-nucleolus region with a low-DAPI signal and clear DAPI-intense (blue) and H3K27m3 (green) and ribonucleoprotein (red) positive margin. **(C)** Nucleolus frequency per experimental group showing significant increases in CTS but no changes in EtOH or EtOH + CTS, related to controls. **(D)** Nucleolar regions lacking both DAPI and euchromatic H3K4m3 central marks in all conditions, evidencing a significant increase in the CTS group. At least 75 cells per condition were analyzed in three independent experiments.

The increased number of nucleoli upon EtOH and/or CTS incubation was another remarkable finding (Figure 8). Nucleoli were identified as non-stained DAPI regions surrounded by a DAPI-intense area, positive to the heterochromatic marker H3K27m3 that recognizes trimethylation of lysine 27 on the histone H3 protein subunit (green), and co-immunopositive to ribosomal subunits (rRNA and ribosomal proteins, red; Kun et al., 2007), as shown in the multiplane view image in Figure 8A. Quantification of the frequency of nucleolus per cell confirmed a greater number of nucleoli than in the controls in CTS (*p = 0.0025*) and a tendency to increase in EtOH and EtOH + CTS (Figure 8B). The Kruskal–Wallis statistics for nucleolus number was 18.62 with *p = 0.0009*.

**FIGURE 9:**
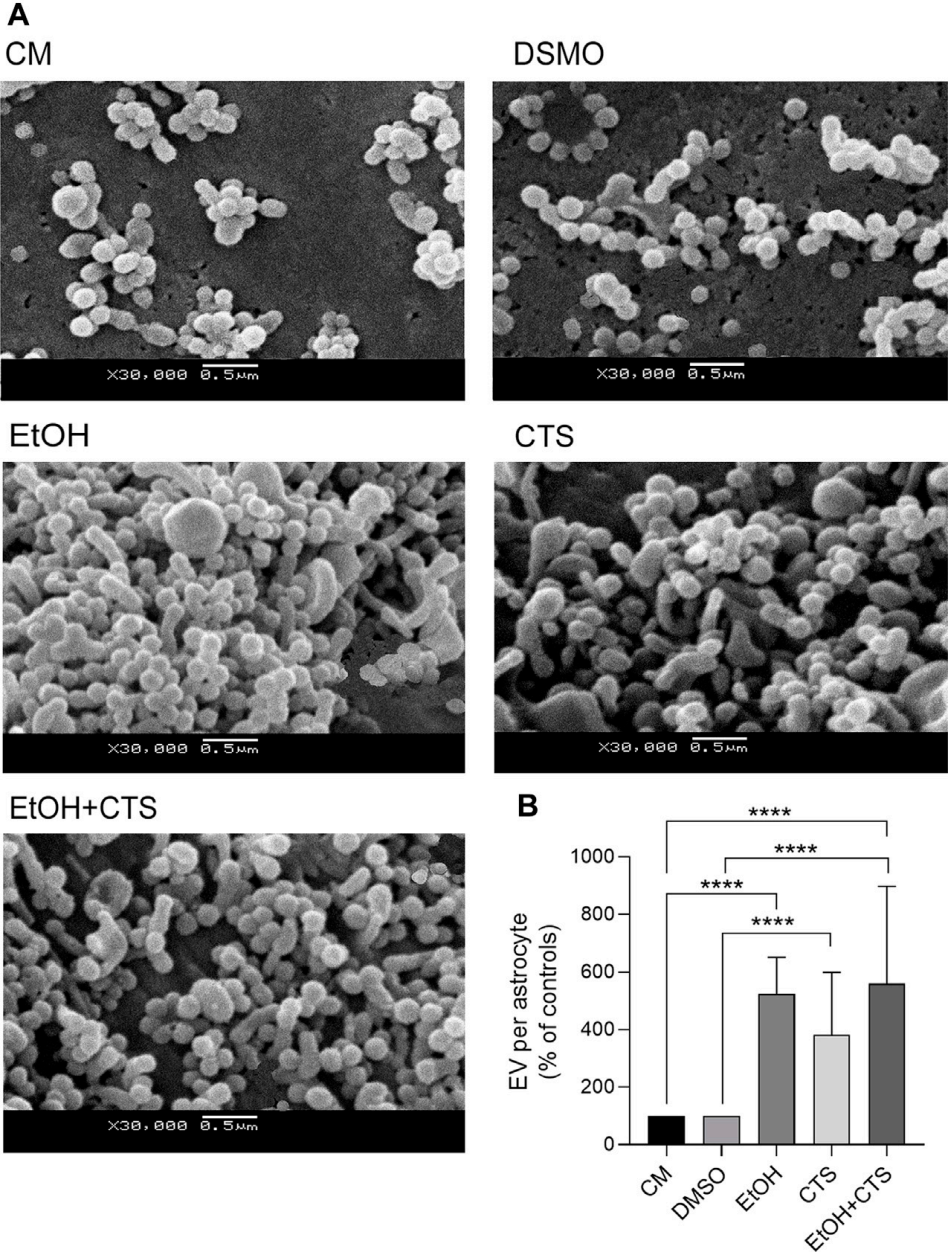
Membrane-attached EV on surfaces of control and EtOH- and/or CTS-exposed astrocytes. **(A)** Representative SEM images showing EV attached to the surface of astrocyte somas from the different experimental conditions. **(B)** EV frequency expressed as controls’ percent in each experimental condition. Significant higher frequency of EV was observed in all treated conditions regarding controls (*p* < 0.0001 in all cases). EV, extracellular vesicle, SEM, scanning electron microscopy.

Finally, modifications in the membrane-associated extracellular vesicles were found upon EtOH and/or CTS incubation, as evidenced by different morphological features and arrangements per experimental condition (Figure 9A). A greater number of membrane-attached vesicles per cell surface was observed in EtOH, CTS, and/or EtOH + CTS when compared with the controls (*p < 0.0001* in all cases, Kruskal–Wallis statistics = 80.64) but without differences among the treated groups (Figure 9B).

## 4 Discussion

### 4.1 Experimental paradigm, strengths, and weaknesses

This work was designed to find out if cultured hippocampal astrocytes would suffer significant and immediate damage to their DNA after 1 h incubation with different concentrations of EtOH or CTS. Dose–response curves generated using the DNA damage sensor, γH2AX, show that astrocyte DNA was significantly damaged at 400 mmol/L EtOH and 1 μmol/L CTS. Interestingly, these concentrations did not trigger a morphological change compatible with immediate astrocyte reactivity, suggesting that the induction of genome damage may be an early event and may participate in the CNS damage cascade, as reported by Kok et al. (2021). Therefore, the major strength of this work was the ability to detect significant DNA damage and immediate cell response in such a short time.

As for weaknesses, the high concentrations of EtOH used are a handicap that we decided to accept in order to obtain immediate DNA damage and be able to analyze the first DDR events. Dose–response studies indicate that for the incubation time used, there is not much space to decrease EtOH working concentrations. However, it is possible to consider performing longer experiments (3–6 h) to evaluate whether DNA damage can be triggered at concentrations closer to those used by problematic alcohol consumers. Although this possibility has a translational advantage, the primary DNA damage and the first DDR events could not be clearly identified from the downstream processes that occur at mid-term upon the injury. In addition, the use of other DNA damage biomarkers different from γH2AX, or its detection with other methods, could confirm the obtained data, enriching this work.

### 4.2 Summary of results

Upon EtOH and/or CTS incubation, the results obtained indicate significant DNA damage, as assessed by H2AX phosphorylation, BER activation, suggested by increased APE1, and cell cycle arrest, as evidenced by decreased nuclear cyclin D1 in CTS. Remarkably, in most of the cases, the EtOH + CTS co-exposure did not elicit distinct results from EtOH or CTS alone. These unexpected results are contrary to the sort of a reinforcing loop between EtOH and CTS levels that the literature suggests since a long time ago (Rivier et al., 1984). Instead, it may imply that the duration of the experimental design was too short to observe the additive effects between both compounds or that each one operates through the same underlying mechanism. The assessment of S100β immunoreactivity, the number of nucleoli, and the frequency of membrane-attached extracellular vesicles evidences an almost immediate cellular response to EtOH and/or CTS. However, GFAP (Figure 2) and phalloidin signal (Supplementary Figure S1) data indicate an absence of significant cell morphology changes at the working concentrations analyzed.

### 4.3 DNA damage and DDR activation markers upon EtOH and/or CTS exposures

H2AX phosphorylation is considered a biomarker of DNA damage because it represents one of the earliest events of DDR, and its nuclear immunoreactivity parallels DNA lesions (Amente et al., 2019). γH2AX is expressed in all of the prototypic neurodegenerative conditions (Merighi et al., 2021), with significant increases in most injured brain regions, such as hippocampus and neocortex, in autopsy samples of Alzheimer’s disease patients (Myung et al., 2008). The levels of γH2AX in glial cells in physiological and pathological conditions are less known, and no previous reports on this parameter were found upon acute EtOH or CTS exposures. The present data show that a single extremely high concentration of EtOH with/without CTS (Flint et al., 2007; Flaherty et al., 2017) elicited significant DNA damage upon 1 h of incubation, as detected by significant and selective nuclear γH2AX immunoreactivity (Figures 3, 4).

DNA damage may underlie some neurological conditions, including those related to age, inherited genetic alterations, or habits (Kok et al., 2021). Previous reports showed that mouse midbrain exposure during 24 h to EtOH generated DNA strand breaks in neurons and glia (Rulten et al., 2008). Regarding astrocytes, these cells accumulated in G0–G1 (Guerri et al., 1990), suffered DNA fragmentation and necrotic death (Holownia et al., 1997), increased proinflammatory markers (Vallés et al., 2004), or decreased mitochondrial activity (Gonthier et al., 2012) upon incubation with EtOH at times ranging from 48 h to 1 week and at concentrations from 20 to 100 mmol/L, respectively. Therefore, the evidence indicates that EtOH induced DNA damage at much lower concentrations than that used in this work but required much longer incubation periods. Conversely, CTS seemed to act faster in accordance with authors who reported that, like other stress hormones, it could elicit DNA damage in cultured murine cells upon exposure times as short as 10 min (Flint et al., 2007; Flaherty et al., 2017) and did not require concentrations at the levels that EtOH needed to elicit significant and comparable γH2AX positive immunoreactivity.

Existing evidence suggests that γH2AX acts not only as a DNA damage marker but also as a first participant in DDR-dependent functions, such as the regulation of cell cycle checkpoints, genomic stability, cell growth, mitosis, and apoptosis (Merighi et al., 2021). In this sense, we found that the M1 Manders coefficient close to 1 in all conditions suggests that the entire γH2AX-positive area colocalized with cyclin D1 (Supplementary Figure S2). This could happen as part of the DDR because γH2AX might recruit cyclin D1 to the damage sites (Rogakou et al., 1998; Fernandez-Capetillo et al., 2003; Kinner et al., 2008) through a series of steps from the DNA damage sensors up to the effectors (Figure 1). This possibility requires to be confirmed by FRET or co-immunoprecipitation assays (Tan and Yammani, 2022). On the other hand, as the M2 Manders coefficient shows, nuclear cyclin D1 signals were not restricted to DNA-damaged areas and spread throughout the whole nucleus, even in the controls (Figure 5); thus, it might have other nuclear functions than those related to DDR (Fu et al., 2004; Tchakarska and Sola, 2020).

Cyclin D1 is a multifunctional protein that has an important role in cell cycle progression by promoting the advancement through G0 to G1 (Figure 1), acting as a mitogenic sensor, and integrating the extracellular mitogenic signals with cell cycle progression (Fu et al., 2004; Tchakarska and Sola, 2020). Cyclin D1 also interacts with chromatin-remodeling factors (p300/CBP and P/CAF) and enzymes (HATs and HDACs) to modify the chromatin structure or with the steroid receptor coactivators (SRC1 and SRC3) to increase the transcriptional activity of the estrogen receptors (McMahon et al., 1999) among other functions. In the context of our experiments, EtOH did not affect the immunoreactivity of nuclear cyclin D1, suggesting no effects on the astrocyte cell cycle or proliferation rate. This is a difference from previous reports that showed EtOH had inhibitory effects on both RNA and DNA synthesis, transcription, and replication in proliferating cells (Rulten et al., 2008) and in brain cortical neuroblasts upon 8–12 h of incubation (Riar et al., 2016). Although in the initial stage, we can speculate that this absence of effects could occur, in part, because EtOH needs more than 1 h to modify astrocyte proliferation in the quiescent level attained upon 24 h of 2% FBS. Conversely, decreased nuclear cyclin D1 upon CTS exposure is consistent with cell cycle arrest. In this sense, Sundberg et al. (2006) showed that CTS decreased the proliferation of embryonic neural stem cells, likely related to cyclin D1 ubiquitin-dependent degradation. It has also been observed that CTS could induce an increase in P27 that inhibits CDK4, which, in turn, is associated with decreases in nuclear CDK/cyclin D1, thus contributing to the cycle arrest and reduced cell proliferation (Jiang et al., 2002; Jirawatnotai et al., 2012).

We also found significant cytoplasmic cyclin D1 immunoreactivity in all conditions, with decreased values relative to controls in CTS but very increased values in EtOH (Figure 5). Cytoplasmic cyclin D1 has been previously reported in astrocytes, likely playing roles in cell–matrix adhesion (Ciapa and Granon, 2018), the regulation of senescence and autophagy (Brown et al., 2012), mitochondrial functions (Sakamaki et al., 2006), or cell migration, which depend on the cyclin D1/CDK4/6 phosphorylation of cytoskeletal proteins involved in cell-shape remodeling (Li et al., 2006; Zhong et al., 2010; Bendris et al., 2015). It is also known that cyclin D1 levels oscillate throughout the cell cycle between the nuclear and cytoplasmic compartments, where, in response to mitogenic signals, the active cyclin D1/CDK4/6 complex enters the nucleus, promoting cell cycle progression, and is then exported to the cytoplasm for its ubiquitin-proteasome degradation (Shan et al., 2009). Although this sort of re-compartmentalization in favor of the cytoplasm can be the case for the EtOH group, the decreased cytoplasmic and nuclear signals in CTS could imply significantly lowered protein levels that could impact the DDR and DNA repair efficacy. In addition, it needed to be confirmed by Western blotting assays.

APE1 is a BER protein (Izumi and Mitra, 1998; Fritz, 2000; Tosolini et al., 2020) with a predominant nuclear expression related to its endonuclease and transcription regulatory roles (Fritz, 2000). However, the APE1 cytoplasmic location was also described, is dynamically regulated (Choi et al., 2016), and is related to the management of oxidative stress (Choi et al., 2016) through the inhibition of both ROS production (Angkeow et al., 2002) and inducible nitric oxide synthase expression (Baek et al., 2016). Cytoplasmic APE1 could also inhibit the induction of proinflammatory mediators and chemotactic cytokines (Joo et al., 2019), even in astrocytes, by impairing TNF-α expression and secretion and/or downregulating NF-κB signaling (Baek et al., 2016), suggesting relevant anti-inflammatory properties (Askalan et al., 2006; Park et al., 2013; Baek et al., 2016). Our results showed significant increases of APE1, both in the nuclear and cytoplasmic compartments of EtOH- and EtOH + CTS-exposed astrocytes, but only in the nucleus upon CTS incubation (Figure 6). Therefore, in the context of our experiments, increased nuclear APE1 immunoreactivity suggests that BER was activated. In addition, in the cases of EtOH and EtOH + CTS, the augmented cytoplasmic APE1 immunoreactivity suggests a modest response that could be associated with cellular antioxidant and anti-inflammatory responses.

### 4.4 Effects of EtOH and/or CTS on the astrocyte status and membrane-EV signaling

We also evaluated if the exposure to extreme EtOH concentrations or CTS elicited changes in the immunoreactivity of the markers of the astrocyte reactive status, GFAP (Figure 2) and S100β (Díaz-Amarilla et al., 2011; Escartin et al., 2021) (Figure 7). GFAP is a protein of intermediate filaments whose aggregation is associated with the typical astrocyte reactivity observed as cell body shrinkage and the emission of significant cell processes. Results obtained at the working concentrations indicate that astrocytes did not show significant cell changes that evidence typical morphological activation. In accordance, only mild increases in GFAP MGV were found upon EtOH and/or CTS challenge.

Concerning S100β, it belongs to the S100 family of calcium-binding proteins localized in the cytoplasm and nucleus of a variety of cells, including astrocytes. S100β binds and competes with GFAP in the astrocyte cytoskeleton (Donato et al., 2009; Yang and Wang, 2015) and is considered a damage-associated molecular pattern (DAMP) with NF-kB signaling as one of its main downstream pathways (Langeh and Singh, 2021). Our results showed increased S100β signals in both the nucleus and the cytoplasm of the EtOH and/or CTS groups related to the controls. This agrees with our previous results showing a predominant nuclear staining in astrocytes from normal tissue sections (Olivera et al., 2008; Olivera-Bravo et al., 2011; Díaz-Amarilla et al., 2011) but a preponderant cytoplasmic presence in reactive astrocytes and anomalous highly neurotoxic and proliferating non-senescent astrocyte phenotypes (Díaz-Amarilla et al., 2011; Jiménez-Riani et al., 2017). S100β results indicate not only increases in the immunoreactivity of nuclear and cytoplasmic compartments suggesting downstream events associated with cascades (Davis and Syapin, 2004; Donato et al., 2009) but also a sort of protein re-localization that seems to be associated with the response to injury as previously described (Díaz-Amarilla et al., 2011; Jiménez-Riani et al., 2017). Such re-localization between different cellular compartments was previously seen in cytoplasmic or membrane proteins found as part of the nucleoskeleton (Philimonenko et al., 2004; Pellegrini and Budman, 2005; McCrea and Gottardi, 2016; Saez and Gonzalez-Granado, 2022) or as nuclear transcriptional coactivators or corepressors (Philimonenko et al., 2004; Pellegrini and Budman, 2005; Hobbs et al., 2016; McCrea and Gottardi, 2016).

Thus, the results obtained indicate a lack of astrocyte reactivity associated with significant morphological changes, but we cannot discard a reactivity-like astrocyte response associated to DDR, as suggested by cyclin D1, APE-1, and S100β results. In line with this possibility, a greater number of nucleoli per nucleus were observed in CTS with a clear tendency to increase in EtOH and EtOH + CTS (Figure 8). Although the augmented nucleolar number was classically linked to an increased likelihood of developing cancer, recent evidence suggests that the nucleolus plays critical roles in many cellular functions that include the response to cellular stressors, maintenance of genome stability, and DNA damage repair (Weeks et al., 2019). Moreover, since cyclin D1 and APE1 were also observed within the nucleoli (visualized as raised rounded regions in astrocyte nuclei in Figures 5, 6), modifications in their immunoreactivity (Figures 5, 6) could, in part, be related to the nucleolus–nucleoplasm interactions recently reported, which would be interesting to address in the future (Pedersen, 2011; Weeks et al., 2019).

In addition, significantly increased number and higher morphological diversity of membrane-extracellular vesicles attached to the astrocyte surfaces upon EtOH and/or CTS exposure (Figure 9) clearly indicate an almost immediate response to such challenges, as early as 1 h later. Although the assessment of EV in this work only included the determination of its density in each experimental condition and its prominent morphological features, the unequivocal differences in both parameters might suggest significant variations in cargoes and changes in the communication repertoire (Lázaro-Ibáñez et al., 2019) associated with CNS damage or with astrogliosis in particular. In this regard, a rising rate of calcium-dependent exocytosis in astrocytes treated with CTS was described (Castellino et al., 1992; Cereseto et al., 2006) as well as an increase in EV secretion with protein cargoes related to inflammation upon EtOH incubation (Ibáñez et al., 2019) and with a wider range of pathological functions described in many neurological conditions (Pegtel et al., 2014).

## 5 Final observations

In summary, here, we described a quick response to either EtOH and/or CTS that includes DNA damage and DDR activation up to downstream effects that may include initial aspects of astrocyte reactivity. Further experiments are necessary to identify the underlying mechanisms and their relevance when assessing astrocyte-mediated effects on neuronal survival and the maintenance of CNS homeostasis.

## Data Availability

The original contributions presented in the study are included in the article/Supplementary Materials; further inquiries can be directed to the corresponding author.
