# Supplementary material for: Astrocyte DNA damage and response upon acute exposure to ethanol and corticosterone
Source: Front Toxicol. 2024 Jan 8;5:1277047. doi: 10.3389/ftox.2023.1277047 (PMC10800529; doi:10.3389/ftox.2023.1277047)
Supplement: Supplementary file 1 [file DataSheet1.pdf]

Supplementary Material

Astrocyte DNA damage response and altered signaling upon acute exposure to ethanol and corticosterone

Ana Laura Reyes-Ábalos, Magdalena Álvarez-Zabaleta, Silvia Olivera-Bravo*†, María Vittoria Di Tomaso†

*** Correspondence:** Silvia Olivera-Bravo: solivera@iibce.edu.uy

† These authors contributed equally to this work and share the last authorship.

Supplementary Figure 1

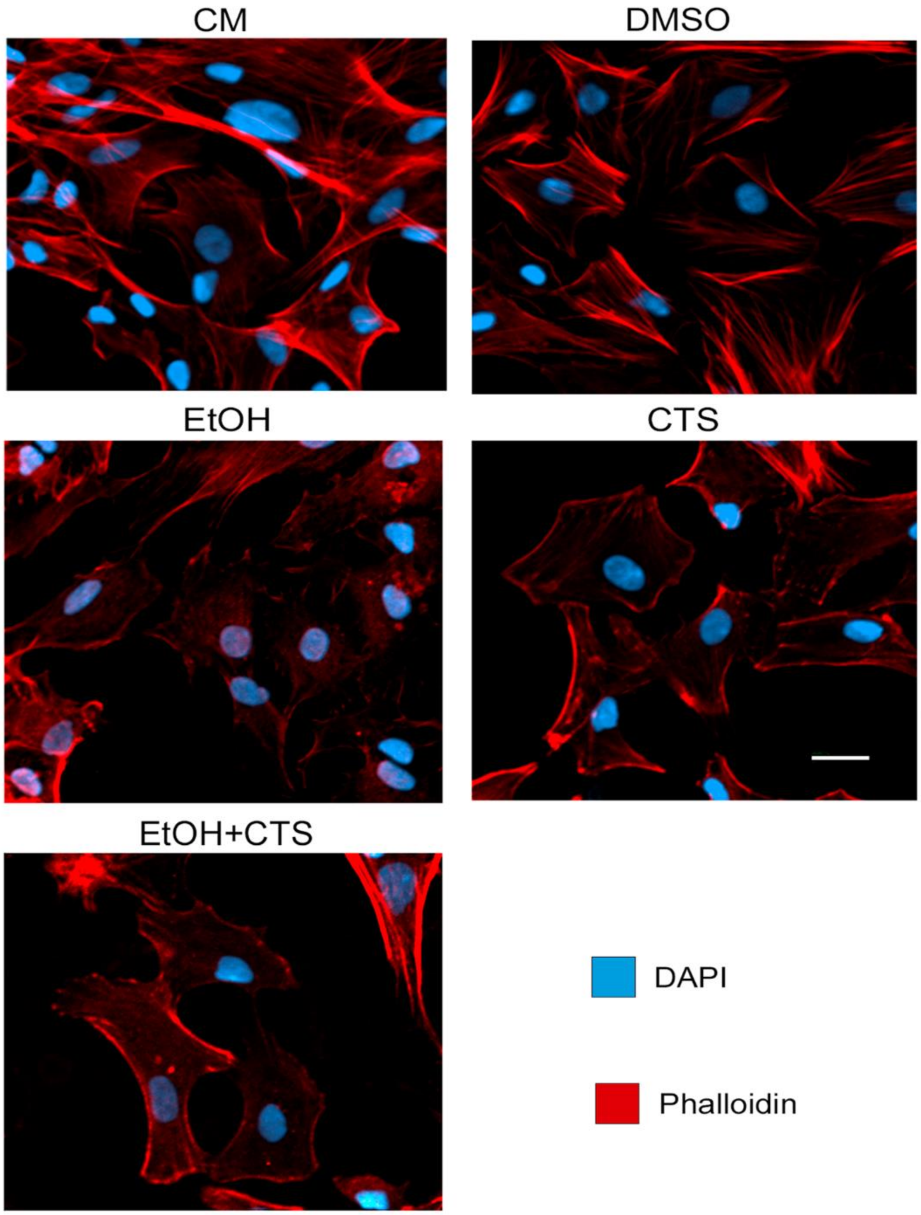

Supplementary Figure 1: Phalloidin labelling of F-actin cytoskeleton of 400 mmol/L EtOH and/or 1μmol/L CTS-exposed astrocytes. Confocal images of Phalloidin signals (red) revealed no changes in cell morphology and preserved F-actin cytoskeleton in all experimental conditions. Nuclei were labeled with DAPI (blue). Calibration bar: 10 μm. One hundred cells were imaged and analyzed in three independent experiments.

Supplementary Figure 2

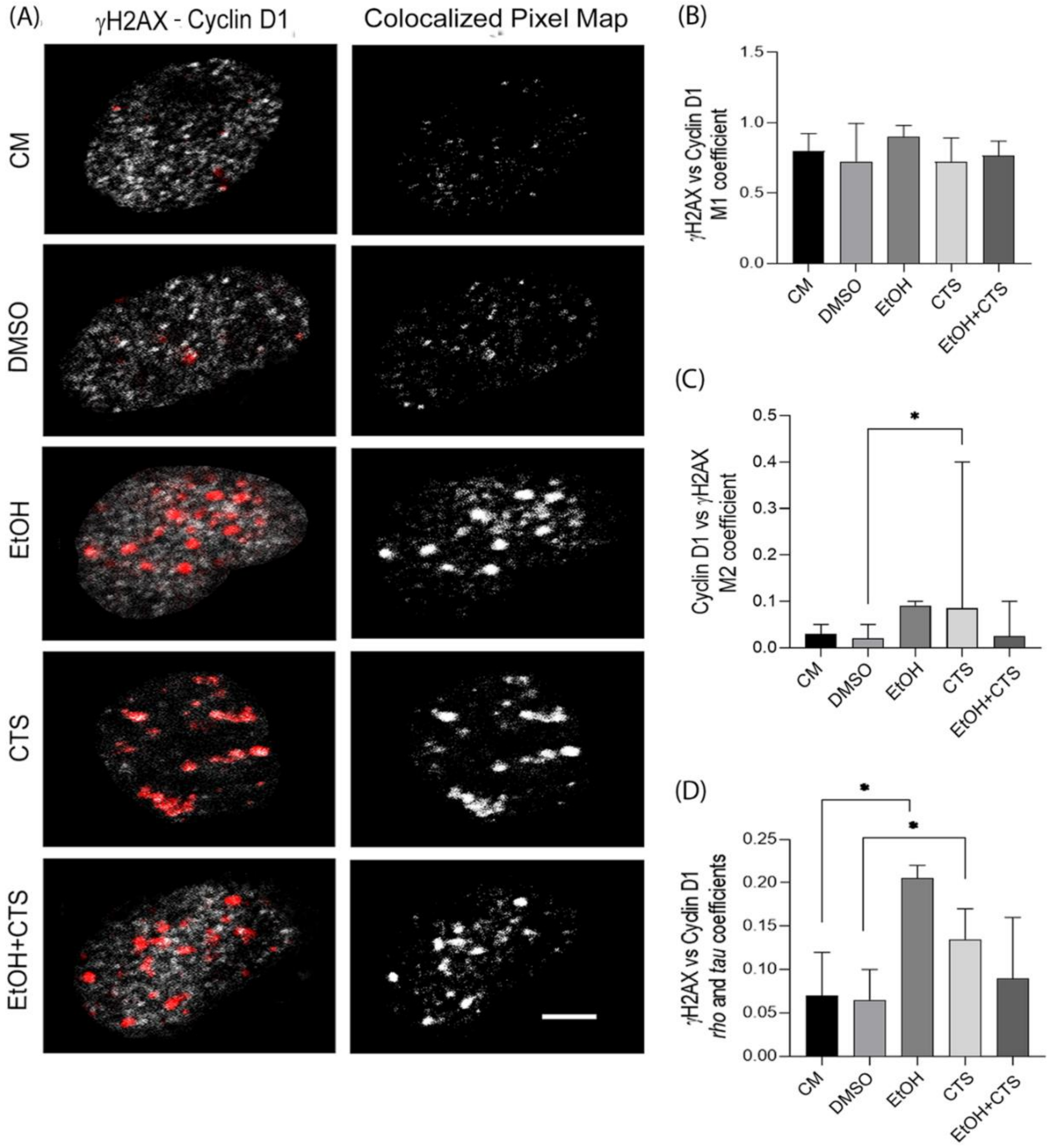

Supplementary Figure 2. γH2AX and Cyclin D1 co-localization upon EtOH and/or CTS incubation. **(A)** Confocal images of individual cells showing γH2AX foci (red) and nuclear cyclin D1 (gray) co-localization (white) on the left. Colocalized pixel map between both markers in all experimental conditions on the right. Calibration bar: 5 µm. **(B-D)** Assessment of colocalization using M1 and M2 co-occurrence **(B, C)** and rho and tau correlation **(D)** coefficients. Interestingly, there were no changes in EtOH and EtOH+CTS but significant increases in CTS, suggesting a higher coupling between both signals. One hundred cells were analyzed in three independent experiments.
